# MTDH is an oncogene in multiple myeloma, which is suppressed by Bortezomib treatment

**DOI:** 10.18632/oncotarget.6610

**Published:** 2015-12-14

**Authors:** Chunyan Gu, Lang Feng, Hailin Peng, Hongbao Yang, Zhenqing Feng, Ye Yang

**Affiliations:** ^1^ Department of Pathology, Nanjing Medical University, 210029, Nanjing, China; ^2^ Basic Medical College, Nanjing University of Chinese Medicine, 210046, Nanjing, China; ^3^ Department of Urology, Beijing Friendship Hospital, Capital Medical University, 100050, Beijing, China; ^4^ Department of Pathology, University of Iowa Carver College of Medicine, 52242, Iowa City, IA, USA; ^5^ Department of Urology, University of Iowa Carver College of Medicine, 52242, Iowa City, IA, USA; ^6^ Department of Internal Medicine, University of Iowa Carver College of Medicine, 52242, Iowa City, IA, USA; ^7^ Department of Laboratory Medicine, Taizhou people's hospital, 225300, Taizhou, China; ^8^ Center for New Drug Safety Evaluation and Research, China Pharmaceutical University, Nanjing, 211198, China

**Keywords:** multiple myeloma, metadherin (MTDH), oncogene, Bortezomib, MMSET (WHSC1)

## Abstract

Metadherin (MTDH) is identified as an oncogene in multiple cancers including breast cancer, bladder cancer and endometrial cancer. However, the function of MTDH in multiple myeloma (MM) is still unexplored. In this study, we disclose that MTDH is an oncogene in MM. This is characterized by an elevation mRNA level of MTDH and chromosomal gain of MTDH locus in MM cells compared to normal samples. Moreover, MTDH expression significantly increased in MMSET translocation (MS) subgroup, one of the high-risk subgroups in MM, and was significantly correlated with MM patients' poor outcomes in Total Therapy 2 (TT2) cohort. Further knockdown of MTDH expression by shRNA in MM cells induced cell apoptosis, inhibited MM cells growth *in vitro* and decreased xenograft tumor formation *in vivo*. Interestingly, opposite to TT2, MM patients with high-MTDH expression showed favorable survival outcomes in the TT3 cohort, while Bortezomib treatment was the major difference between TT2 and TT3 cohort. Furthermore we proved that Bortezomib suppressed pre- and post-transcription levels of MTDH expression of MM cells *in vitro* and *in vivo*. Finally, our studies demonstrated that MTDH is a transcriptional gene of MMSET/NFκB /MYC signaling in MM cells, which is inhibited by Bortezomib treatment.

## INTRODUCTION

Multiple myeloma (MM) is the second most common malignancy residing in the bone marrow, which exhibits gene expression changes and cytogenetic abnormalities commonly influencing the Immunoglobin locus [1–4]. Many of these abnormalities like T(4;14) MMSET/FGFR3 translocation, 1q21 gain et al. promote the development of drug resistance and aggressive disease. To better recognize the molecular basis of MM, Shaughnessy JD Jr et al. categorized MM patients into 8 subgroups based on Gene expression profiling (GEP), including CD1 and CD2 subgroup with CCND1 and CCND3 translocation, hyperdiploidy (HY) group, myeloid-like group (MY), low bone disease (LB) group, MMSET/FGGR3 spike group (MS), MAF/MAFB (MF) spike group and proliferation (PR) group [5, 6]. MF, MS and PR subgroups comprised high-risk MM with worse outcome than other 5 groups and were designated as low-risk groups. To date, although the utility of novel high-dose chemo-therapeutic reagents combining with autologous stem cell transplant (ASCT) confer survival advantage, MM patients still surfer from relapse and die of MM eventually. Thus continued research to identify innovative modes of action in MM and to develop its specific inhibition method are still in urgent requirement.

Metadherin, also known as astrocyte elevated gene-1 protein (AEG-1) or protein LYRIC, is a protein encoded by the MTDH gene in humans. MTDH is significantly amplified and acts as an oncogene in multiple cancers including breast cancer [7], melanoma [8], malignant glioma et al [9]. Increased MTDH promotes HRAS induced tumor-promoting effects [10], facilitates breast cancer metastasis [11], and activates NFκB transcription through accumulating nuclear translocation of p65 in Hela cells [12]. However the function of MTDH in multiple myeloma (MM) is still not elucidated and requires further exploration.

In this study, we first compared the MTDH expression in MM cells with normal control cells, classified MTDH expression in different MM subgroups, and correlated MTDH with MM patient outcomes in TT2 and TT3 cohorts. We also discovered that Bortezomib suppressed MTDH expression in both MM cell lines and primary samples. Finally, we disclosed the mechanism by which Bortezomib inhibited MTDH expression in MM.

## RESULTS

### Increased MTDH correlates with poor survival in MM

**Figure 1:**
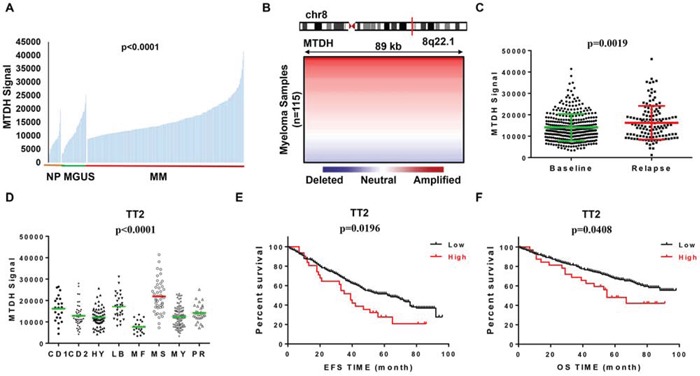
MTDH levels are correlated with poor survival in newly diagnosed myeloma patients **A.** MTDH expression of normal plasma cells (NP, n=22), monoclonal gammopathy of undetermined significance cells (MGUS, n=44) and myeloma patient plasma cells (n=351) in GEP dataset. **B.** Array-based comparative genomic hybridization analysis of the MTDH locus at human chromosome 8q22.1 in 115 primary MM samples. **C.** MTDH expression in 130 relapsed MM patients and 351 newly diagnosed MM patients from TT2 GEP cohort. **D.** A box-plot showed MTDH expression in 8 MM subgroups of TT2 cohort. **(E & F)** Kaplan-Meier analysis on the event free survival **E.** and overall survival **F.** of MM patients in TT2 cohorts based on the MTDH expression.

To trace the role of MTDH in MM, we examined MTDH expression in normal plasma (NP), monoclonal gammopathy of undetermined significance (MGUS, a pre-MM disease) and myeloma cells using our gene expression profiling (GEP) database [13]. Notably, MTDH expression increased considerably from NP, MGUS, to MM samples (p<0.0001, one-way ANOVA) (Figure 1A). Further analysis of array-based comparative genomic hybridization (aCGH) data collected from 115 MM patients revealed that the MTDH locus is frequently amplified in these MM patient samples (Figure 1B) [14] implying MTDH may also behave as a tumor-initiating gene in MM. In addition, MTDH expression significantly increased in the relapsed MM patients from Total Therapy 2 (TT2) cohort compared to newly diagnosed patients in the same cohort (Figure 1C) [15]. Figure 1D confirmed this and showed that increased *MTDH* was particularly prevalent in the MMSET-activating translocations subgroup (MS) compared to other 7 subgroups in TT2 cohort, which is one of the subgroups with the poorest prognosis in MM (p<0.0001, one-way ANOVA, Fig. 1D) [5]. To correlate with clinical parameters, MTDH expression represented an independent factor associated with characteristics like IgA isotype (p<0.05), hypodiploid (p<0.05), and especially 1q21 amplification by FISH analysis (p<0.01), which is acknowledged as a poor diagnosed marker in MM (Table 1) [16]. Above all, MM patients bearing high MTDH expression suffered poor clinical outcomes relative to low-MTDH-expressing patients in TT2 (Total Therapy 2) cohort and as shown in Figure 1E and 1F elevated MTDH expression is linked to significantly shorter response duration of both event free (EF) and overall survival (OS) respectively. Thus we may propose that MTDH acts as an oncogene in MM as well.

**Table 1:** The Correlation of MTDH Expression and Clinical Characteristics in TT2

Characteristics	High MTDH	Low MTDH	p Value
	(%, n = 32)	(%, n = 319)	
Age at least 65 years	28.1	21.3	NS
Female sex	46.9	42.9	NS
White race	93.8	88.1	NS
IgA isotype	43.8	23.8	<0.05
CRP at least 4.0 mg/l	9.4	5.6	NS
β2-Microglobulin at least 4.0 mg/l	43.8	33.5	NS
Hemoglobin less than 10 g/dl	21.9	25.4	NS
Albumin less than 3.5 g/dl	50	35.1	NS
Creatinine at least 2.0 mg/dl	6.3	11.6	NS
MRI focal bone lesions, at least three	46.9	57.4	NS
LDH at least 190 IU/l	37.5	33.5	NS
Hyperdiploid	12.5	19.1	NS
Hypodiploid	28.1	14.1	<0.05
Amplification of 1q21	65.6	43.9	<0.01

### Decreased MTDH expression induced MM cell growth inhibition *in vitro* and *in vivo*

**Figure 2:**
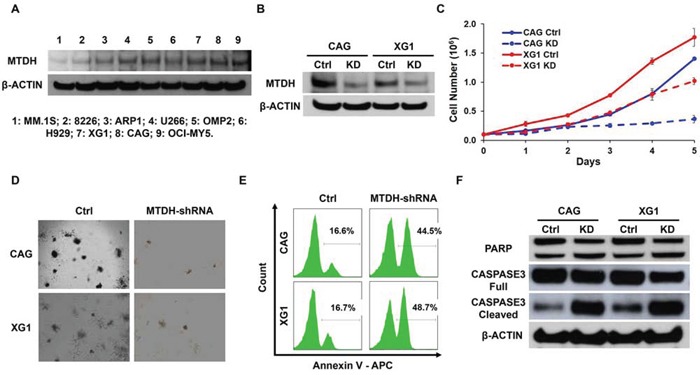
Decreased MTDH expression induces MM cell growth inhibition *in vitro* **A.** Western blot on MTDH expression in 9 MM cell lines. **B.** MTDH expression in CAG and XG1 cells were detected by western blot after MTDH-shRNA transfection. **C.** Cell numbers of MTDH-knockdown (KD) and Control (Ctrl) cells from CAG and XG1 were counted with a hemocytometer for 5 days. **D.** Clonogenicity evaluation on long-term cellular growth rate of the Ctrl and MTDH^KD^ CAG and XG1 cells. **E.** Flow cytometry showed the apoptosis cells labeled by Annexin V after MTDH-shRNA lentivirus was transfected for 48h in CAG and XG1 cells. **F.** Western blot of CAG and XG1 Ctrl and KD cells on the PARP and Caspase 3 expression.

To determine MTDH functions as an oncogene/driver gene in MM rather than a sequential phenomenon based on previous data in Figure 1, we functionally knocked down MTDH expression in MM cells by using lentiviral shRNA transfection. Since MTDH is widely expressed at the mRNA level in primary patient MM cells indicated in Figure 1, we further confirmed its protein expression by Western blot and found that all the 9 MM cell lines ubiquitously expressed MTDH (Figure 2A). Then we suppressed MTDH expression in CAG and XG1 cells by lentiviral shRNA particles. Immunoblotting was recruited to verify the efficiency of shRNA. As shown in Figure 2B, MTDH expression was remarkably decreased at protein level in MM cells transfected with MTDH-shRNA (KD) compared to the control (Ctrl). To explore the role of MTDH on MM cellular growth, cell numbers of KD and Ctrl cells were counted daily using trypan blue. After cultured for 5 days, MTDH-KD MM cells displayed significantly lower cell growth rate compared to the Ctrl cells in both CAG and XG1cells (Figure 2C). The growth inhibition effect of MTDH was further confirmed by colony formation assay. As shown in Figure 2D, significant reductions of colony number were observed in MTDH-shRNA transfected cells relative to its corresponding control cells in the assay. The diminished growth rate of MTDH-KD cells was attributed to the increased apoptotic cell death detected by flow cytometry using Annexin V antibody after MTDH-shRNA was transfected for 48h (Figure 2E). The MTDH-shRNA induced apoptosis in MM cells was also examined by western blot, in which the intensity of cleaved PARP and Caspase 3 bands were higher than the control cells (Figure 2F). These results suggest MTDH expression is critical for promoting MM cell growth *in vitro*.

**Figure 3:**
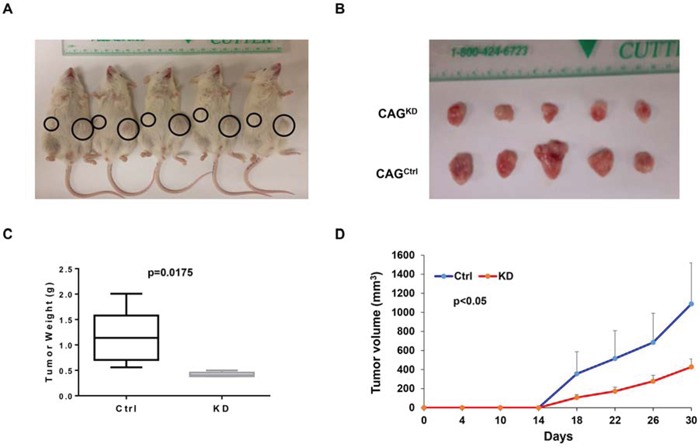
Downregulation of MTDH inhibits myeloma xenografts in NSG mice **A.** Tumor growth in NSG mice xenografts with CAG Ctrl and MTDH^KD^ cell in each flank (n=5) on Day 30 of the cells inoculation. **B.** Tumors dissected from NSG mice in Panel A. **C.** Mean weight of CAG Ctrl and MTDH^KD^ xenografts on day 30 post myeloma cell injection. **D.** Time course of tumor growth in NOD/SCID mice received CAG Ctrl and MTDH^KD^ cell in each flank (n=5).

To extend our findings to *in vivo* study, we xenografted CAG^KD^ and CAG^Ctrl^ cells subcutaneously into the two-side flank NSG mice respectively (n = 5). Tumor diameters were measured twice per week to evaluate the growth rate of the CAG^KD^ and CAG^Ctrl^ xenografts. In 5 of 5 cases, the tumors derived from CAG^KD^ cells were visibly smaller than their CAG^Ctrl^ counterparts (Figure 3A & 3B). The average weight of CAG^KD^ tumors (0.42 g) was 37% lower than the control tumors (1.14 g; Figure 3C). Time course regression analyses of growth rates demonstrated that the CAG^KD^ tumors volume outstandingly lagged behind their corresponding partner CAG^Ctrl^ tumors (Figure 3D). These results indicate that genetic knockdown of MTDH inhibits myeloma *in vivo*.

### MTDH expression was suppressed by Bortezomib treatment in MM

**Figure 4:**
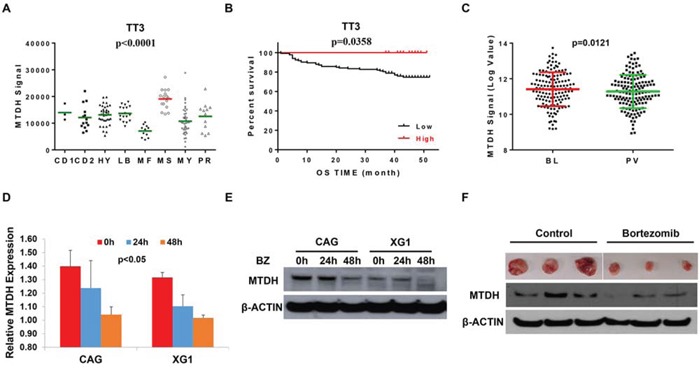
Bortezomib suppresses MTDH expression in MM **A.** The distribution of MTDH levels among 8 multiple myeloma subgroups in TT3 cohort by GEP. **B.** Kaplan-Meier curve of MM overall survival according to the MTDH expression in TT3 cohort. **C.** A box-plot of MTDH expression in 142 paired MM cells before and after Bortezomib treatment for 48h. **(D & E)** qPCR **D.** and western blot **E.** detection on MTDH expression in MM cells treatment with Bortezomib for 24h and 48h in CAG and XG1 cells. **F.** Western blot on the MTDH expression in xenograft tumors derived from NSG mice treated with Bortezomib or PBS control respectively (n=3).

MTDH was identified as oncogene in multiple cancers including MM which denotes that MTDH is an intriguing therapeutic target for cancer treatment, nevertheless the MTDH inhibitors still await for further investigation. In this study, we found that MTDH expression showed similar distribution among the patients in TT2 and TT3 cohorts, highest MTDH expression in MMSET subgroup (Figure 4A), however, patients with high MTDH expression in TT3 cohort displayed contradictory outcomes compared to patients in the TT2 cohort (Figure 4B), and enjoyed more optimistic overall survival than low-MTDH-expressing patients. As Bortezomib treatment is the major difference between the TT2 [17] and TT3 [18] strategy of newly diagnosed MM patients, we speculate that MTDH might be a therapeutic target of Bortezomib. Using publicly available GEP datasets, we proved this hypothesis and showed that MTDH expression in MM cells from 142 newly-diagnosed patients was significantly decreased after Bortezomib treatment for 48h (PV) compared to the corresponding MM cells collected before treatment (BL) in the TT3 cohort (p=0.0121, (Figure 4C) [13, 18]. This result was stressed by study of Van Ness BG et al., in which they reported Bortezomib treatment consistently reduced MTDH expression in MM1S, U266 et al. four MM cell lines at two time points, 16h and 24h by GEP [19] (Figure S1). We further verified the effect of Bortezomib on MTDH expression in MM cell lines by qPCR and western blot. MM cell lines, CAG and XG1, were treated with 2 nM Bortezomib and collected at 24h and 48h after treatment. Compared to non-treatment control cells, mRNA (Figure 4D) and protein level (Figure 4E) of MTDH were decreased by Bortezomib treatment consistently. Xenograft tumors (n=3) from NSG mice treated with Bortezomib or PBS control were dissected and western blot was performed to examine MTDH expression after Bortezomib treatment *in vivo*. Compared to control tumors, Bortezomib-treated tumors comprised reductive amounts of MTDH protein (Figure 4F). Our results strongly support that Bortezomib suppresses MTDH expression in MM.

### Bortezomb treatment suppresses MTDH through MMSET/NFκB/MYC signaling pathway in MM

**Figure 5:**
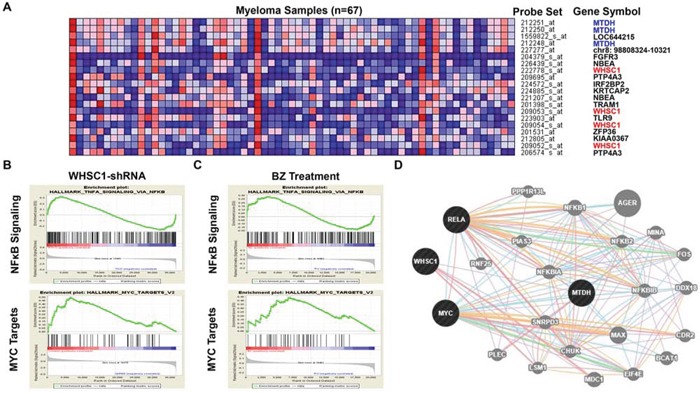
MTDH expression and Bortezomib treatment are associated with MMSET/NFκB/MYC signaling in MM **A.** The top 20 GEP neighbor genes (GEP probe sets) correlated with MTDH in MM analyzed by Multiple Myeloma Portal. **B. & C.** GSEA analysis of MM cells transfected with MMSET-shRNA and MM patients' samples before and after Bortezomib treatment for 48h. **D.** Genetic network of MTDH, MMSET, NFκB and MYC generated with the GeneMANIA online tool.

To investigate the mechanism of Bortezomib treatment suppressing MTDH expression in MM cells, we evaluated the top 20 neighbors genes/probe sets that are co-expressed with MTDH in the gene expression dataset published by Carrasco DR et al [20] (Figure 5A). Among the top 20 gene probe sets co-expressing with MTDH, 4 WHSC1/MMSET probe sets were listed suggesting MTDH may be activated by MMSET transcription in MM. This hypothesis was supported by the previous finding that MTDH enriches significantly in MMSET subgroups compared to other subgroups in both TT2 and TT3 cohorts. Then, we continued to query for the signaling shared by MMSET and Bortezomib treatment using Gene set enrichment analysis (GSEA). We explored the Molecular Signatures Database (MsigDB) gene sets based on Scramble control vs MMSET-KD MM cells dataset [22, 23] and MM cells before and after Bortezomib treatment dataset respectively. Intriguingly, we found that both knockdown of WHSC1/MMSET and Bortezomib treatment in MM cells were highly related to NFκB signaling and MYC signature in the GSEA analysis (Figure 5B & 5C). Genetic interaction network of MTDH, WHSC1/MMSET, NKκB and C-MYC was integrated and generated with the help of the GeneMANIA online tool, which predicts that MTDH is activated by NKκB and C-MYC signaling pathway (Figure 5D).

### MTDH is transcribed by MMSET/NFκB/MYC signaling in MM

**Figure 6:**
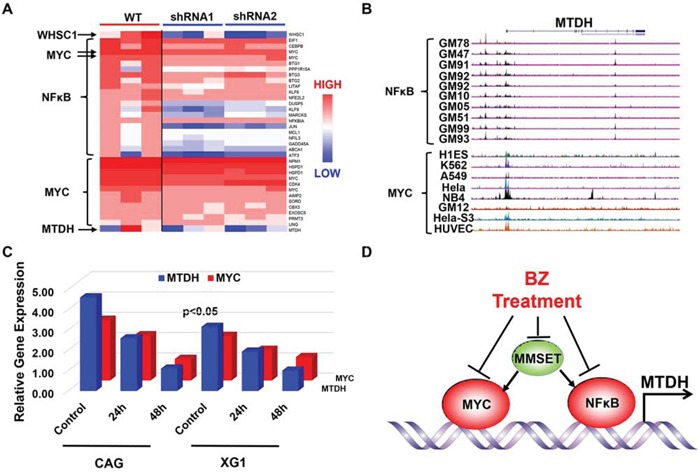
NFκB and MYC directly transcribe MTDH expression in MM **A.** Heatmap showed the significantly differentiated genes from NFκB and MYC signal before and after MMSET was knocked down in KMS11 MM cells. **B.** NFκB and MYC chromatin occupancy pattern at the MTDH locus revealed by ChIP-Seq analysis in multiple cells. **C.** NFκB inhibitor suppressed MYC and MTDH expression in mRNA level shown by qPCR. **D.** An illustration for working model of Bortezomib inhibition on MTDH in MM.

Microarray analysis revealed that knockdown MMSET expression by shRNA remarkably reduced MTDH expression, NFκB signaling, and MYC signature genes described by the MSigDB gene sets in GSEA (Figure 6A). MYC is also a downstream gene of NFκB signaling according to the MSigDB gene sets and is decreased by the MMSET shRNA as well. Then we explored the chromatin immunoprecipitation sequencing (ChIP-Seq) database from the University of California Santa Cruz (UCSC) for chromatin occupancy patterns at the MTDH, which showed a significant footprint of NFκB/P65 binding on the promoter region of MTDH in 10 of 10 B-lymphocytes. The promoter region of MTDH was also occupied by MYC in multiple cancers including Hela, MCF-7 et al. (Figure 6B) suggesting that NFκB/MYC directly transcribes MTDH expression and promotes oncogenetic activity in cancer cells. Consistently, NFκB inhibitor, SN50 Peptide (2μM), inhibited MTDH and MYC transcription in a time-dependent manner detected by qPCR (Figure 6C). Summarily, Borbetzomib treatment suppresses MTDH expression in MM through inhibiting MMSET/NFκB/MYC signaling (Figure 6D).

## DISCUSSION

In this study, we found increased MTDH expression in MM cells compared to normal plasma control cells and MGUS cells (Figure 1A) and chromosomal gain of MTDH in most of MM patients by CGH array (Figure 1B). Impressively, elevated MTDH expression was detected in relapsed MM patients relative to newly diagnosed patients (Figure 1C), and patients in MMSET subgroup, a high-risk MM subgroup, bear the highest MTDH expression than other 7 subgroups (Figure 1D) suggesting a poor outcome of high MTDH expressing patients. More importantly, patients with high MTDH expression suffered from worse survival than MTDH low-expressing patients in both event free and overall survival (Figure 1E & 1F). To further validate the role of MTDH in MM, we knocked down MTDH expression in MM cell lines using lentiviral shRNA and the results showed that decreased MTDH induced MM cellular growth inhibition (Figure 2C), clonogenicity reduction (Figure 2D), apoptosis *in vitro* (Figure 2E & 2F) and tumorigenicity suppression *in vivo* (Figure 3). This data was also supported by the finding that the positive relation of MTDH with Ki67, and negative correlation with Capase-3 by histology staining from other cancers [24–27].

Based on these studies, we conclude that MTDH is an oncogene in MM as well, which makes MTDH as an attractive therapeutic target for MM. Inspiringly, contradict to TT2 cohort, high MTDH expression is associated to good outcome in TT3 cohort while Bortezomib treatment is the major difference between both two cohorts (Figure 4B). Thus, we infer MTDH may be a therapeutic target for Bortezomib. Further study proved this and showed that Bortezomb treatment inhibited MTDH expression in MM cell lines *in vitro* (Figure 4D & 4E), in xenograft mice (Figure 4F) and MM patients' samples *in vivo* (Figure 4C).

To query the mechanism underlying the Bortezomib treatment induced MTDH reduction, we explored MTDH neighbor genes using Myeloma Portal, and MMSET/WHSC1 was identified as the most relevant gene with MTDH (Figure 5A). Chng WJ et al. reported that Bortezomib reduced MMSET expression in MM while MMSET directly bound with NFκB and promoted NFκB transcription [21]. Moreover, GSEA analysis on three independent database showed that both MMSET-shRNA and Bortezomib treatment suppressed NFκB and MYC signaling genes (Figure 5B) and Chip-seq analysis from Santa Cruz revealed that MTDH is a downstream gene of NFκB and MYC transcription in multiple cells (Figure 6B). Therefore, we proposed that Bortezomib reduced MTDH expression through inhibiting MMSET/NFκB/MYC signaling cascade. We further validated this proposal by treating MM cells with NFκB inhibitor, which hindered MTDH expression in a time-dependent manner. Chng WJ et al. also presented that Borbezomib treatment decreased p65 in KMS11, KMS18 and KMS28BM MM cells [21]. In addition, Bortezomib is also able to block proteasome-dependent p100 conversion to p52 resulting in inhibition of non-canonical NFκB activity suggesting the importance of Bortezomib on NFκB signaling in MM. Meanwhile MYC also transcribes MTDH expression and stimulates oncogenetic activity in cancer cells including MM. The oncogenetic activity of MYC is altered by Bortezomib treatment to induce cancer cell death by enhancing expression of the pro-apoptotic BCL2 family members NOXA and BIM [33, 34].

In summary, we identified the oncogenetic function of MTDH in MM and pointed out that Bortezomib was a therapeutic reagent for MTDH through inhibiting MMSET/NFκB/MYC transcription (Figure 6D). Our study provided a preclinical framework for MTDH inhibition derived from MM to other cancers.

## MATERIALS AND METHODS

### Cell lines and cell culture

Human MM cell lines, CAG and XG1, were cultured in RPMI 1640 medium (Gibco, Grand Island, NY) containing 10% heat-inactivated fetal bovine serum (FBS) (Gibco), penicillin and streptomycin (P/S) solution (100 μg/mL, Sigma, St. Louis, MO) in humidified 95% air and 5% CO_2_ at 37°C.

### Reagents

MTDH antibody from Invitrogen (Grand Island, NY) was kindly provided by Dr. Xiangbing Meng (Department of Obstetrics and Gynecology, University of Iowa) and β-ACTIN (Catalog number: #4967) was from Cell Signaling Technology (Danvers, MA). Bortezomib was purchased from Selleck Chemicals. (Houston, TX). NFκB inhibitor, SN50 Peptide, (17493) was obtained from Cayman Chemical Company (Ann Arbor, MI).

### Quantitative Real time-PCR assays (qPCR)

Total RNA was extracted using RNeasy RNA isolation kit (Catalog number: 74104, Qiagen, Germany) and reverse transcribed with the SuperScript III RT kit with oligo dT primers (Catalog number: 18080-051, Invitrogen, Carlsbad, CA). Quantitative Real-time PCR primers were from Integrated DNA Technologies (Coralville, IA). Standardization of samples was performed with the endogenous control, β-ACTIN. Fold changes were calculated with the ΔΔCt method. Sequences of primers are as following: MTDH (5′- GGA GTC AAG ACA CTG GAG ATG C -3′ and 5′- GGG TTG ATT ACG GCT AAC ATC CC -3′) and β-ACTIN (5′- CAC CAT TGG CAA TGA GCG GTT C -3′ and 5′- AGG TCT TTG CGG ATG TCC ACG T -3′).

### Western blots

Western blots were utilized to measure the protein levels in MM cells. Briefly, cells were firstly lysed in Mammalian Cell Extraction Kit (Catalog number: K269-500, Biovision, Milpitas, CA). Around 20 μg protein per sample was loaded to SDS-PAGE using 4%-12% polyacrylamide gels prior to the nitrocellulose membrane transfer. Membrane was blocked with 5% non-fat dry milk in Tris buffered saline (TBS) containing 0.05% Tween-20 (TBST), and then incubated overnight at 4°C with primary antibodies. Western bands were visualized with HRP-conjugated secondary antibodies and SuperSignal West Pico (Pierce, Rockford, IL). Membrane was subsequently stripped and re-probed for β-ACTIN as controls.

### Soft agar clonogenic assay

Clonogenic formation was performed by seeding 10,000 MM cells in 0.5 mL 0.33% agar supplemented with RPMI 1640 medium with 10% FBS in 12-well plate. The cells were incubated at 37°C with 5% CO_2_ and fed by the same medium for 1-2 week, twice per week. The plates were imaged and colony numbers were calculated by Image J.

### Lentivirus expression vector system

MTDH lentiviral based gene silencing particle (sc-77797-V) were was purchased from Santa Cruz Biotechnology (Dallas, Texas). MM cells were transfected and cells were selected using Puromycin according to the protocol. Transfected efficiency was verified by Western blot.

### Gene expression profiling (GEP) and data analysis

GEP, using the Affymetrix U133 Plus2.0 microarray, was performed as previously described [13, 35]. Microarray data and outcome data used in this study have been deposited in the NIH Gene Expression Omnibus [13, 19, 36, 37].

### A xenograft myeloma mouse model

MM cells (2 × 10^6^) were injected subcutaneously into the abdomen of 6-8 weeks′ NOD. Cg-Rag1 (NSG) mice (Jackson laboratory, Bar Harbor, Maine) (n = 5). After 10 days, Bortezomib (1 mg/kg, IP) treatment was started and injected twice per week. Tumor burdens were monitored by tumor volume. The mice were sacrificed by CO_2_ asphyxiation when subcutaneous tumors reached 20 mm in diameter.

### Statistical analysis

The MM patients' survival data were plotted by Kaplan-meier curve and analyzed using log-rank test. Multiple groups (n≥3) were analyzed with one-way ANOVA, and other paired values were analyzed by two-tailed Student's t-test and expressed as mean ± SD. A p<0.05 was considered as significant.

## SUPPLEMENTARY FIGURE


