# Supplementary material for: MTDH is an oncogene in multiple myeloma, which is suppressed by Bortezomib treatment
Source: Oncotarget. 2015 Dec 14;7(4):4559–69. doi: 10.18632/oncotarget.6610 (PMC4826226; doi:10.18632/oncotarget.6610)
Supplement: Supplementary file 1 [file oncotarget-07-4559-s001.pdf]

SUPPLEMENTARY FIGURE

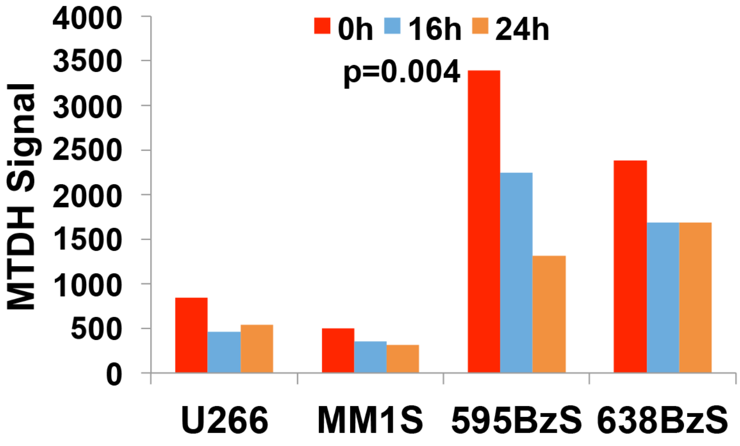

Supplementary Figure S1: The expression of MTDH in 4 MM cell lines before and after Bortezomib treatment detected by GEP (P =0.004 by one-way ANOVA).
